# Round and flat zygomatic implants: effectiveness after a 1-year follow-up non-interventional study

**DOI:** 10.1186/s40729-022-00412-8

**Published:** 2022-04-01

**Authors:** Carlos Aparicio, Waldemar D. Polido, James Chow, Rubén Davó, Bilal Al-Nawas

**Affiliations:** 1 Zygomatic Unit at Hepler Bone Clinic, ZAGA Center, Roman Macaya, 22-24, 08022 Barcelona, Spain; 2 International Teaching Scholar, Indiana University School of Dentistry, Indianapolis, USA; 3 Oral and Maxillofacial Surgery, Indiana University School of Dentistry, 1121 W. Michigan Street, Indianapolis, IN 46202 USA; 4 ZAGA Center, Indiana University, Bloomington, USA; 5 ZAGA Center Hong Kong, Associated Brånemark Osseointegration Center, 1901-1905, 1912-1913, The Center, 99 Queen’s Road Central, Hong Kong, Hong Kong SAR; 6 ZAGA Center, Alicante, Spain; 7 Dentistry and Maxillofacial Department, International Vithas Medimar Hospital, c/ Padre Arrupe, 20, 03016 Alicante, Spain; 8 Department for Oral and Maxillofacial Surgery, Plastic Surgery, University Medical Center of the J. Gutenberg University Mainz, Augustusplatz 2, 55131 Mainz, Germany

**Keywords:** Zygomatic implants, ZAGA implants, ZAGA-Flat, ZAGA-Round, ZAGA, ORIS criteria

## Abstract

**Introduction:**

There are few zygomatic implants (ZI) designs available. The objective of this non-interventional study was to report the effectiveness of two new site-specific ZI, selected and placed following the zygoma anatomy-guided approach (ZAGA).

**Materials and methods:**

Consecutive patients presenting indications for rehabilitation using ZI were treated according to ZAGA Concept recommendations. Implants were immediately loaded following the manufacturer’s instructions. Success criteria regarding prosthetic offset, rhino-sinus status, soft tissue condition, and implant stability were additionally used as outcome parameters.

**Results:**

Twenty patients were followed for a period of 12 to 28 months (average 18.8 months). Ten received 2 ZI plus regular anterior implants; One received 3 ZI plus regular implants and nine received 4 ZI. In total, 59 ZI were placed, 34 (58%) Straumann ZAGA-Flat design, and 25 (42%) ZAGA-Round. Forty-nine percent of the sites were classified as ZAGA-4 type and 27% as ZAGA-2. Four patients (20%) presented discontinuities of the sinus–nose floor before surgery and 15 patients (75%) presented previous sinus opacities. All implants bar one reached more than 45 N.cm of insertion torque. No surgical complications were observed. After 1 year, the modified Lund–Mackay score was negative in 17 patients. Seventeen sites in 11 patients exhibited decreased opacity when pre-surgical imaging was compared to 1-year post-surgical CBCT. All implants and prostheses remained stable and in function.

**Conclusions:**

The study concluded 100% implant/prosthesis survival rates and low complication levels. Within the limitations of the sample and observation period, results suggest that even in cases of extremely resorbed maxillae (as per cases in this study), ZAGA-Flat and ZAGA-Round ZI are viable treatment options when restoring atrophic maxillae following the ZAGA protocol.

## Introduction

For decades, zygomatic implants have been employed to rehabilitate atrophic maxillae and to reconstruct congenital and acquired maxillary defects [1]. Currently, multiple zygomatic implants are successfully implemented to manage patients with extremely resorbed maxillae [2–4]. Recently, Davó et al. conducted the first randomized controlled trial comparing zygomatic implant treatment to conventional implant treatment in augmented maxillae [5]. Results showed that immediately loaded zygomatic implants had fewer prosthetic complications, higher implant survival rates, shorter treatment periods, and better patient acceptance.

Previously described systems for the installation of zygomatic implants, such as the Original Procedure [1, 6, 7], the Slot Technique [8], or the Extra-Sinus Approach [9–13] support a unique surgical process to be applied for all patients. However, different morphologies of the edentulous maxilla have been identified, both between individuals and intra-individuals [14]. Therefore, the use of the same osteotomy type in all situations (i.e., “window” plus intra-sinus entrance, “slot” plus crestal osteotomy, or “extra-maxillary” pathway) will frequently generate complications. These include bulky prosthetic constructions, impaired hygiene, sinus complications, and or soft tissue dehiscence.

**Fig. 1:**
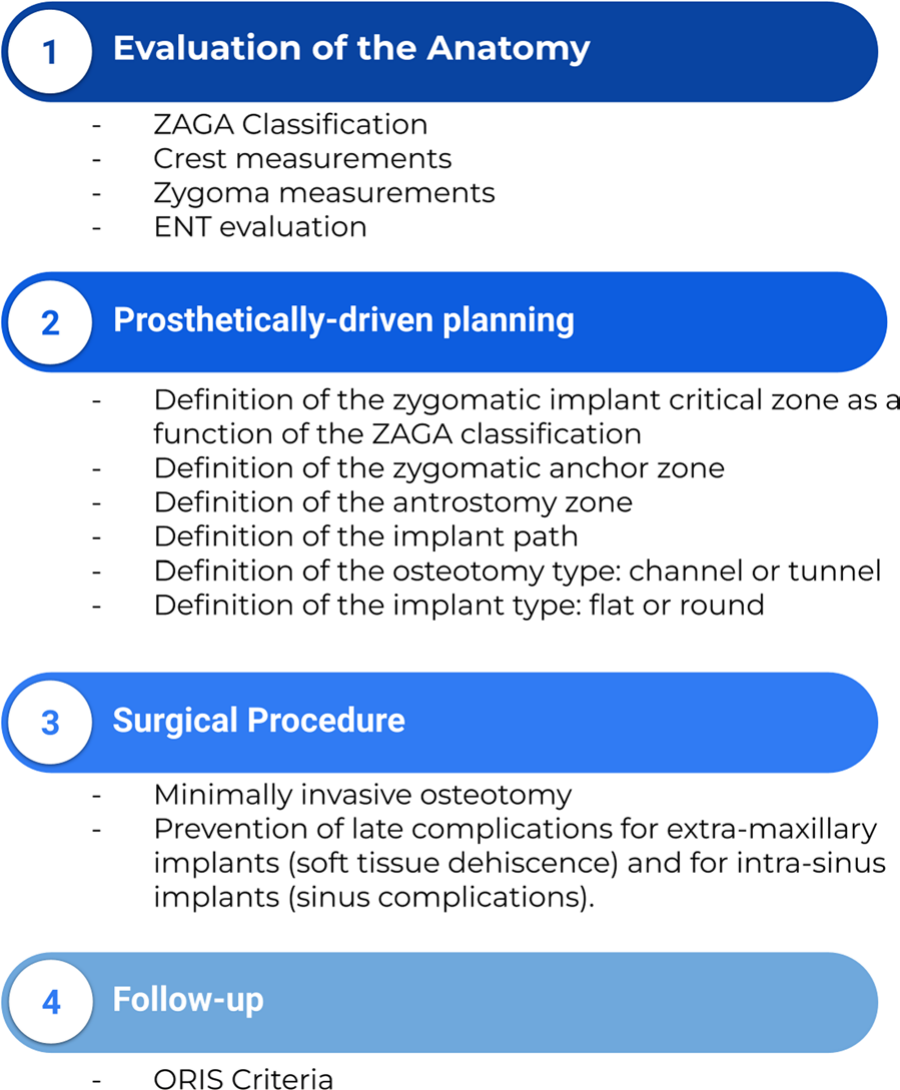
ZAGA diagram

The Zygomatic Anatomy-Guided Approach (ZAGA) [14, 15] was described as a guideline to assist in the selection of the correct technique for each implant pathway depending on the anatomy of the patient to prevent complications. The concept aims to provide the surgeon with a decision-making protocol for both the implant path and surgical technique when planning. Thus, the ZAGA Concept is a “patient-specific therapy” as it adapts the procedure to the anatomy of each patient (Fig. 1). The determination of the implant path depends on prosthetic, bio-mechanic, and anatomic criteria. While using the ZAGA Concept to plan and execute, the implant path may be intra-sinus, extra-sinus, or reach multiple intermediary positions using the maxillary wall as an additional source of anchorage. Usually, no initial window or slot is opened at the lateral wall of the maxillary sinus. This approach has been described and used in different studies and publications [16–19].

The ORIS criteria of success for ZI [20] have been used to holistically follow up on each patient. Using the ORIS criteria, comparative study results, between the original technique and the ZAGA Concept, consistently show less traumatic osteotomy; better implant stability, and bone-to-implant contact; together with improved bone sealing around the implant neck. Additionally, the rate of late sinus complication dramatically decreases, and greater anatomic rehabilitation is achieved [21].

Because the zygomatic implant path involves the atrophic alveolar bone, the maxillary wall, and the zygomatic bone, it presents greater irregularities than the implant path in a regular implant indication. While there are numerous regular implant designs that can be used on the residual alveolar bone, the selection of the implant design, i.e., length, diameter, shape, etc., should be determined in accordance with residual bone quality or quantity. Despite many patients presenting with anatomical differences in the complex formed by the atrophic alveolar remanent, the anterior maxillary wall, and the zygomatic bone, very few zygomatic implants designs are available [22].

Randomized controlled trials provide strong insights into the safety and effectiveness of a product. However, these studies may not be realistically performed in the case of patients presenting severely atrophic maxillae. Due to the high variability among patients, the statistical power required to design a randomized controlled trial with such variability makes it extremely complex and lengthy to implement. Also, inclusion and exclusion criteria may not precisely replicate the condition of a dental office and can result in varying success rates [23, 24]. For these reasons, it has been argued that the results of clinical studies may lack external validity and might not be representative of actual outcomes seen in a general population, thus indicating the need for complementary observational studies [23]. Although there is more freedom in treatment decisions with an observational study, the systematic documentation of the use of each device, and the resulting clinical outcomes provide an overall assessment of how the device performs in clinical practice over time [24].

The objective of this non-interventional study is to report and evaluate the safety and performance of two “site-specific” zygomatic implant designs in a series of patients in different centers following the ZAGA protocol. Observations were carried out over a minimum of one-year post-loading, using the ORIS criteria.

## Material and methods

### Study population

This is a prospective non-interventional study. The study subjects were patients who presented with indications for zygomatic implants and agreed to participate in the study. After an evaluation of the patient’s general health status, and a comprehensive assessment of the maxillary defect in relation to the final prosthesis, the criteria used for the indication of ZI placement were mainly radiological. Therefore, guidelines followed the checklist for radiological evaluation as published by Aparicio et al. [25]. The number of zygomatic implants was decided by the residual anatomy of the maxilla. According to Bedrossian et al. [26], when it was possible to place regular implants (longer than 10 mm) in areas between canine abutments, but was not possible to treat posterior molar and premolar areas without using bone grafts, then placement of two posterior zygomatic implants, plus regular implants in the anterior area, was indicated. In cases where alveolar bone height was less than 4 mm in the molar and premolar areas of the maxilla or less than 10 mm in the anterior area, the placement of four zygomatic implants was indicated. This same indication was used when the alveolar architecture of the maxillary anterior areas was considered unfavorable for placement of regular implants without additional augmentation or bone regeneration procedures. Patients with sufficient maxillary bone that could be rehabilitated using conventional straight or tilted implants were excluded [19]. Subjects were considered smokers when exceeding 10 cigarettes per day. Since this was a non-interventional study, no other specific inclusion/exclusion criteria existed. All the implants were installed by the same surgeon (CA) with extensive experience in zygomatic implant surgery. Five different clinicians with high experience in full arch rehabilitation and implant maintenance participated in the restorative treatment of the patients.

### Implant designs

**Fig. 2:**
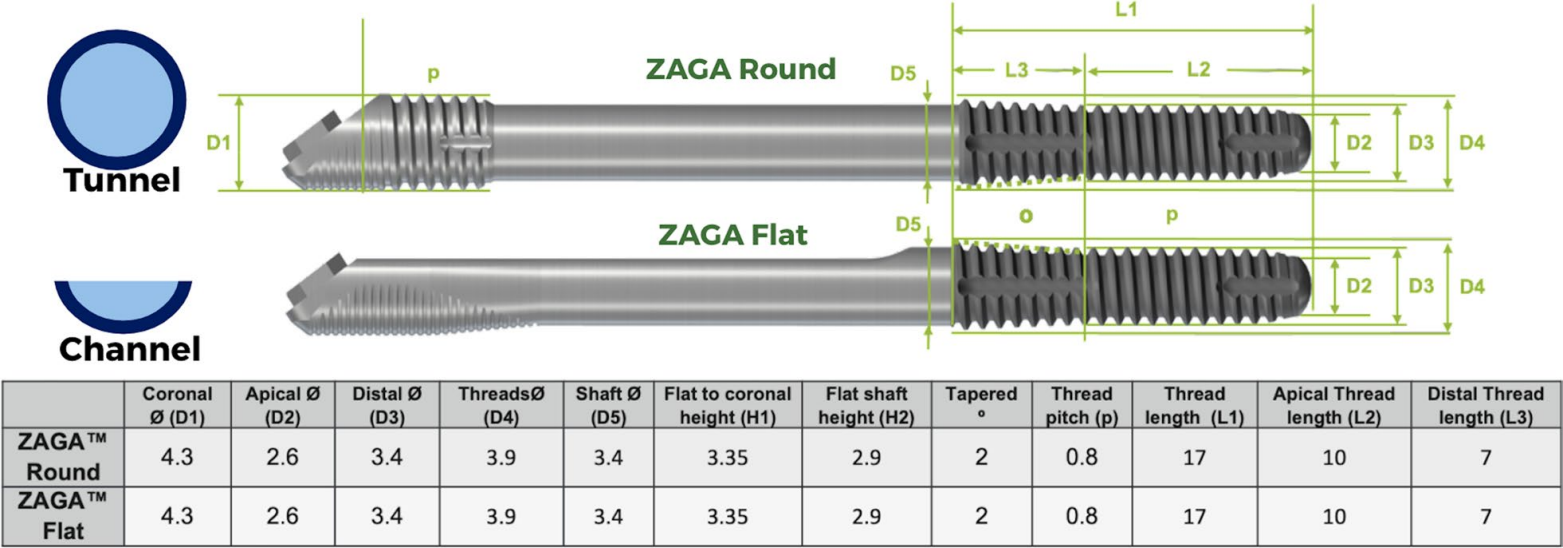
Technical characteristics, similarities, and differences between the two types of zygomatic implants are graphically shown. Straumann ZAGA-Flat has a circular segment section, whereas Straumann ZAGA-Round has a circular one. Both zygomatic implants share unique features of having a tapered design and narrow diameter

According to the manufacturer’s surgical and prosthetic procedures, the Straumann® Zygomatic Implant System includes two different designs: the Straumann® ZAGA™ Round with circular section and the Straumann® ZAGA™ Flat with a circular segment section on its body and coronal parts. Both incorporate a head angle correction of 55°. Apical regions are roughened, threaded and tapered for both implant designs. Both have smooth machined surfaces at the body and coronal regions. This implant system uses a fixture mount having the same diameter as the coronal part of the implant head (4.3 mm). Both implants are indicated for immediate loading when good primary stability is achieved, and with appropriate occlusal loading (Fig. 2).

### Study protocol

All patients underwent an oral and radiological examination including a maxillary CBCT before zygomatic implant placement. The scan was used for digital planning using DTX Studio Implant software (Nobel Biocare AG). Virtual implant osteotomy trajectories were established using the zygoma anatomy-guided approach (ZAGA). Indeed, the ZAGA Concept uses a prosthetically driven zygomatic implant trajectory adapted to each patient’s anatomy. Therefore, implant trajectories varied from externalized to intra-sinus [14, 16]. The ZAGA Concept includes a minimally invasive osteotomy, the goals of which have been described by Aparicio et al. [6, 16–18] and are shown in Table 1.

**Table 1:** Influence of implant design on ZAGA minimally invasive osteotomy goals

Goal	Implant feature	Results
Place the implant head at the optimal dental position using a prosthetically driven implant trajectory	Implant axis 55º correction	Easier ideal prosthetic positioning Implant-to-abutment junction is not located at the zygomatic implant critical zone (ZICZ). This eliminates the possibility of bone resorption due to eventual bacterial leakage
Achieve optimal anterior–posterior distribution of the implants	Reduced apical diameter	The reduction of the apical diameter increases the possibility of divergent positioning of the implant shafts, thus improving the final AP distribution
Achieve maximal implant primary stability	Apical tapered self-cutting design	If a conservative osteotomy is performed, the difference between the diameter of the last drill and the progressive section of the implant achieves greater primary stability
Preserve as much bone as possible at the maxillary wall and alveolar bone	Threads and/or micro-threads are incorporated at the implant head/neck level	Threads, together with implant stability, facilitate osteointegration and bone stability
Maximize bone-to-implant contact (BIC) along the length of the whole implant. This includes alveolar, maxillary wall, and zygomatic bone	The tapered apical design experiences an increased diameter at the level of the implant neck. The drilling protocol shows a difference between implant diameter and last drill diameter (0,5 mm at the apical level increasing to 1,4 mm at the implant neck/head)	Increased BIC along the entire length of the implant
Achieve complete sealing of the osteotomy by the implant body	two types of implant section, round and flat	The clinician may decide which design would better adapt to the performed osteotomy
Protect sinus integrity at the implant head/neck level to prevent late sinus–oral communication	Implant-to-abutment connection is not located at the ZICZ Threads and/or micro-threads at the head neck level Machined surface at implant head and body	No bacterial leakage and subsequent bone resorption is expected at the ZICZ Threads together with stability and alveolar bone contact will enhance the possibility of osseointegration If a soft tissue recession occurs, machine-surfaced implants will maintain surrounding soft tissue health better than a rough-surfaced implant
Prevent soft tissue dehiscence	A design presenting a flat surface is available	By facing the flat surface against the soft tissue, any eventual compression of its vessels is diminished, thus decreasing the possibility for dehiscence

The subjects' treatment generally included diagnostics, surgery, immediate prosthesis delivery within 24 h, and follow-up after implant loading. The follow-up included 2 week and 4-month visits after immediate loading of the screw-retained provisional prostheses, along with necessary visits for final prostheses delivery. Afterward, a follow-up control visit was scheduled every 6 months. The first annual control included a CBCT. Medical and dental history was recorded at baseline, and complications were recorded throughout the entire study period. All aspects of Implant planning and placement were recorded. These included the type, size, and location of each implant, insertion torque, abutment type, and length.

### Clinical protocol

**Fig. 3:**
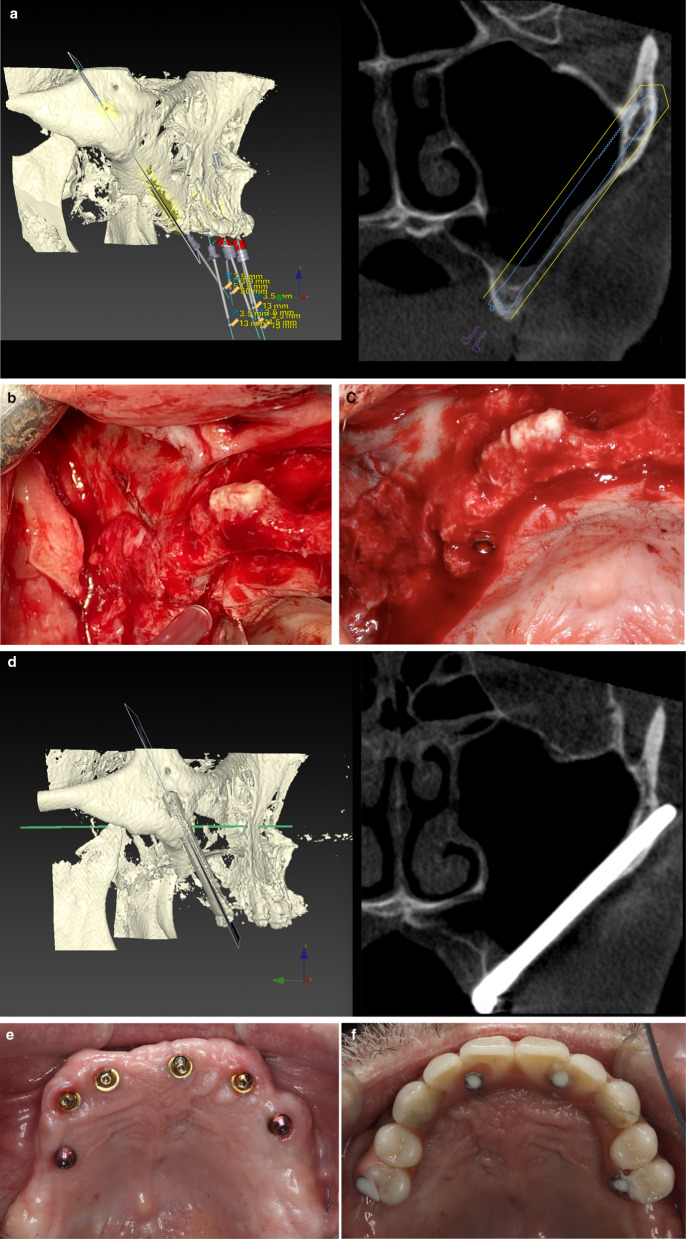
**a** CBCT cut showing the chosen virtual planning for a zygomatic implant in a maxilla classified as ZAGA type 0. The finding of adequate alveolar dimensions together with a flat maxillary wall converts the intra-sinus implant path into the first election. **b** Clinical picture illustrating the “tunnel type” osteotomy entrance and the external pencil guideline that has been drawn previously to the osteotomy. **c** Occlusal clinical picture illustrating the final position of the Straumann ZAGA-Round implant head, totally closing the circular osteotomy. **d** CBCT cut after one year of implant placement. Implant stability together with implant neck osseointegration into adequate bone architecture is interpreted as the reason for long-term sinus transparency maintenance. **e** Occlusal clinical picture showing soft tissue stability at one-year follow-up. The chosen intra-sinus path in a maxilla presenting enough residual alveolar bone and a flat maxillary wall is not associated with soft tissue problems nor with non-anatomic prostheses. (Patient treated in collaboration with Drs. Peter and Madalina Simon, ZAGA Center Stuttgart, Germany.) **f** Occlusal view of the provisional prostheses. Note the favorable emergence of the prosthetic screws

**Fig. 4:**
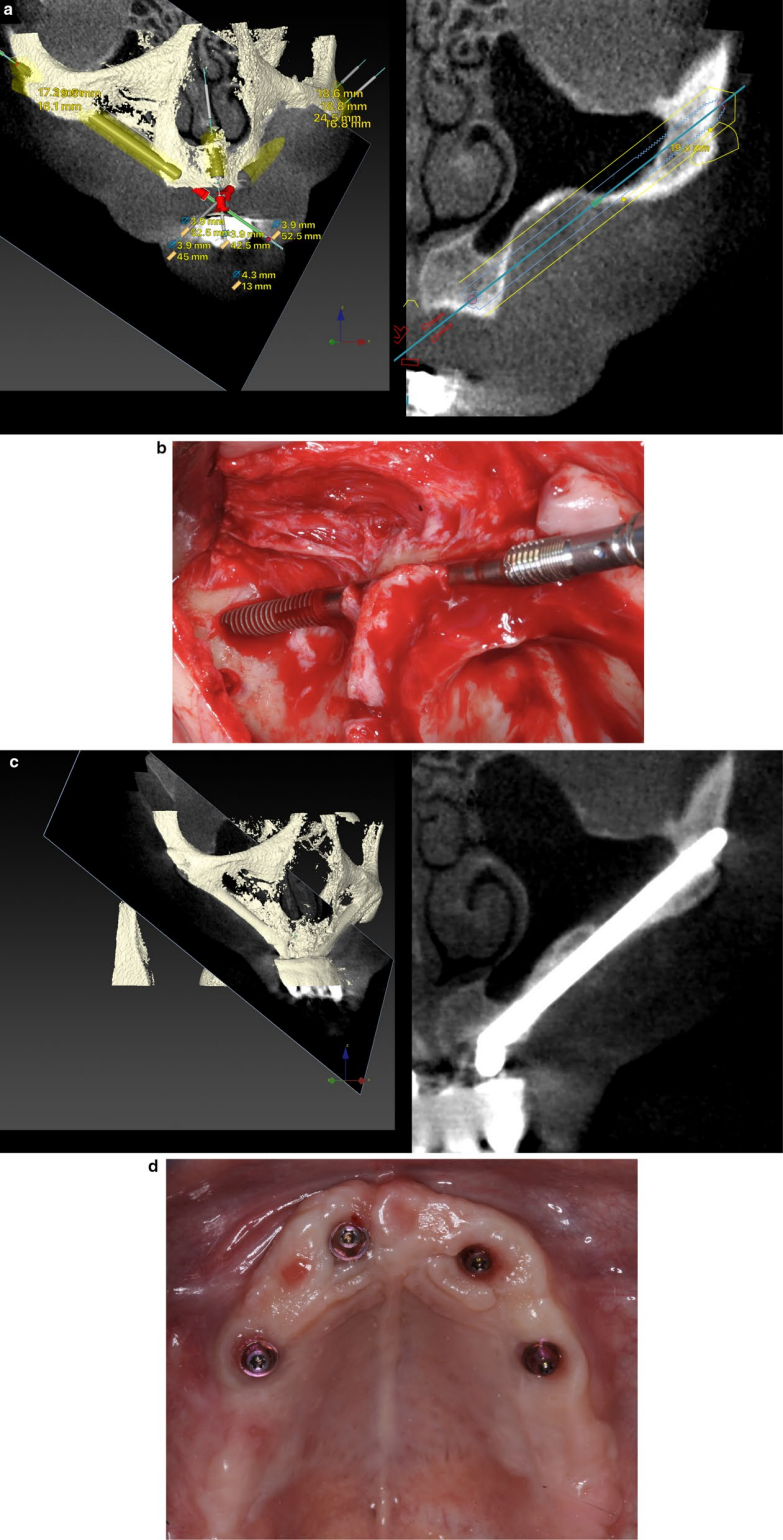
**a** Oblique CBCT cut illustrating the planned implant path in a ZAGA Type 3 anatomy, starting from the lateral incisor/canine area until reaching the zygomatic bone. Note that the antrostomy zone (AZ) is planned far from the zone where the implant meets the alveolar bone or Zygomatic Implant Critical Zone (ZICZ). **b** Clinical picture illustrating a Straumann ZAGA-Round implant being screwed into the osteotomy planned in a. The tapered implant tip has a rough surface, whereas its body has a turned one. **c** The oblique cut of the DTX Studio Implant software (Nobel Biocare AB) is showing 3-D and 2-D images of the Straumann ZAGA-Round zygomatic implant one year after its placement. Sinus transparency is facilitated by the maintenance of sinus lining integrity at the level of the ZICZ and the placement of the AZ is located far from the ZICZ. Note that remains of alveolar bone have been maintained buccally to the implant neck to facilitate soft tissue fibers attachment preventing dehiscences. **d** Occlusal image of the patient represented in **a**–**d** taken 24 months after the surgery. Soft tissue maintenance is facilitated by several factors like adequate incision design, the use of site-specific implants, respect for alveolar bony remains maintenance, and correct placement of the ZICZ. (In collaboration with Drs. Pedro Guitian and Elena López ZAGA Center Vigo Spain and Drs. Edmon Bedrossian and Sepehr Zarrine.)

The volume and architecture of the alveolar/basal process and the curvature of the anterior maxillary wall were crucial factors for establishing the coronal implant position. When the bone architecture at the nasal/sinus floor level was considered sufficient to house the implant neck (that is, ≥ 4 mm high and 6 mm wide) in an adequate alveolar architecture, all attempts were made to place the implant using a tunnel-shape osteotomy (Figs. 2, 3). The term “tunnel osteotomy” is used because the alveolar osseous entry point has a floor, lateral walls, and a more or less complete roof. When a tunnel osteotomy was chosen, the sinus membrane was perforated at the time of completion of the antrostomy. The objective was to achieve osseointegration at the neck level also to seal the sinus entrance in the long term using: (1) a stable zygomatic implant with (2) a suitable threaded neck section (3) bordered by enough bone at the coronal entry, (4) stabilized by adequate apical zygomatic anchorage; and (5) connected to a rigid prosthesis that provides adequate masticatory load distribution. The tunnel osteotomy was also employed in ZAGA Type 3 cases when the alveolar bone adopts a triangular, buccally inclined, profile and the maxillary anterior wall was concave (Figs. 2, 4). The tunnel type of osteotomy is typically used on ZAGA types 0, 1, and 3 and has a circular section entry point that will be sealed by a round implant. Thus, a Straumann® ZAGA™ Round zygomatic implant was chosen.

**Fig. 5:**
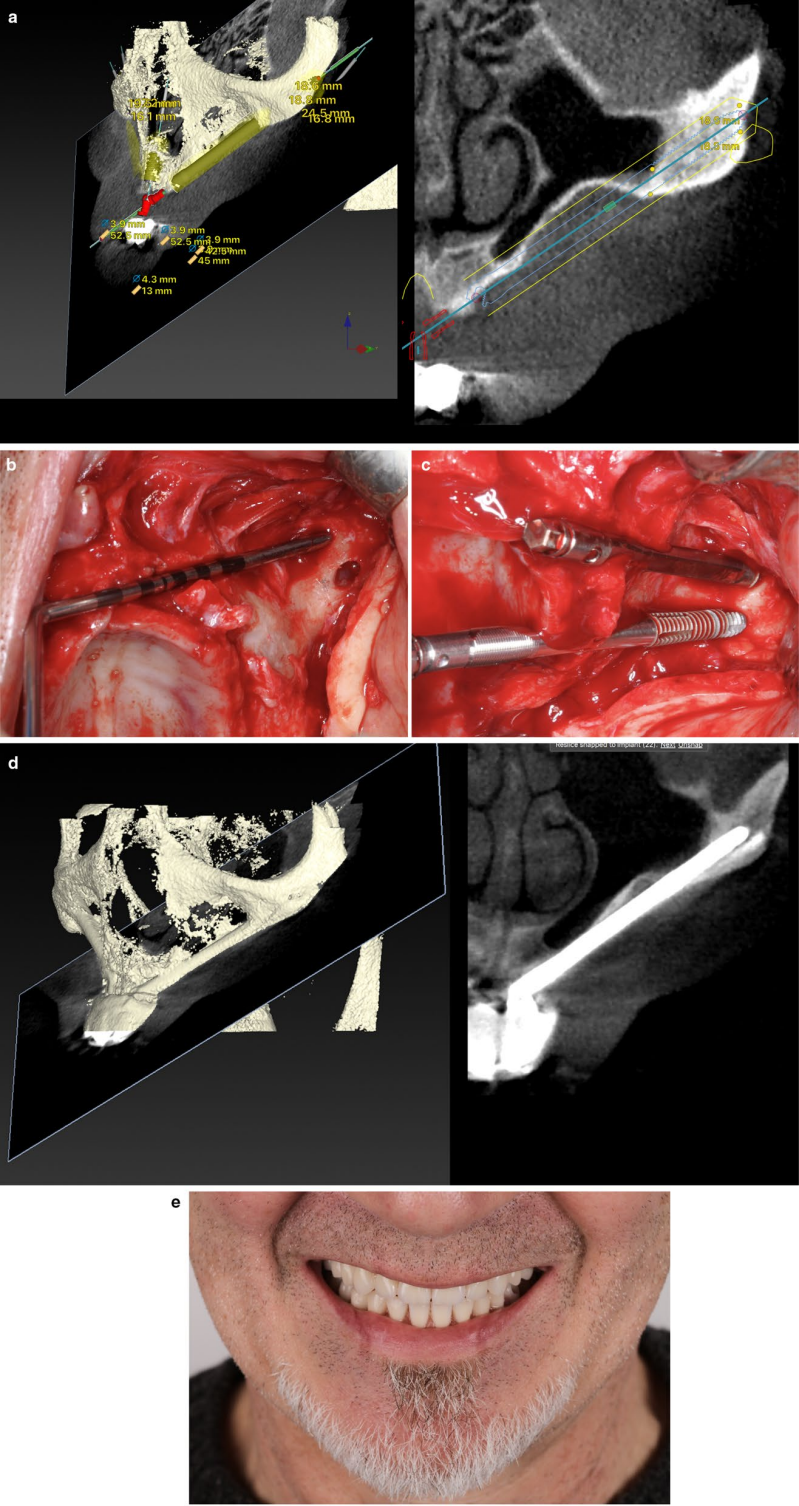
**a** Virtual planning for an anterior zygomatic implant in an anterior maxilla ZAGA Type 4. The amount of residual alveolar bone is insufficient to host the implant. In order to respect sinus lining integrity at the ZICZ, the implant path has been buccally shifted. **b** Clinical picture illustrating the depth measurement of the minimally invasive ZAGA osteotomy. Note the aiming for an under-preparation preserving as much bone as possible on both sites alveolar and maxillary wall. **c** The anterior ZAGA-Flat implant is already in place. Note the micro-threads on the implant neck opposite the flat surface. Apical self-cutting flutes help the tapered apical portion of the implant to achieve full primary stability. **d** 3 and 2-D images of the implant described in (**a**)–(**e**) one year later. Sinus integrity has been respected. **e** Esthetic prosthetic result of the upper rehabilitation and trial of the lower jaw. (Rehabilitation performed by Drs. Pedro Guitian and Elena López ZAGA Center Vigo Spain.)

When inadequate residual bone architecture at the crestal level (< 4 mm of thickness) was measured, the coronal osteotomy was buccally shifted to prevent future sinus or nasal–oral communication/fistula (Figs. 2, 5a–d). Implant beds were designed to be carved as much as possible into both the buccal alveolar and maxillary wall bone with the limits of sinus lining integrity. This osteotomy type is known as a “channel osteotomy and is noted when lateral walls and floor are present but no roof” Because the Straumann® ZAGA™ Flat design had a circular segment section, it was chosen to fit the channel and present its flat surface against the soft tissue. As such, it was expected to reduce vascular soft tissue compression.

After implant surgery, an immediate screw-retained provisional prosthesis was placed in all patients. The design of the immediate prostheses was decided by each center, in accordance with the desired treatment plan for the subject. A non-smoking period of at least 2 weeks and a soft diet for 3 months were strongly recommended. Following a period of at least 4–6 months, a more elaborated screw-retained prosthesis was delivered (Fig. 5e).

### Outcome measure

**Fig. 6:**
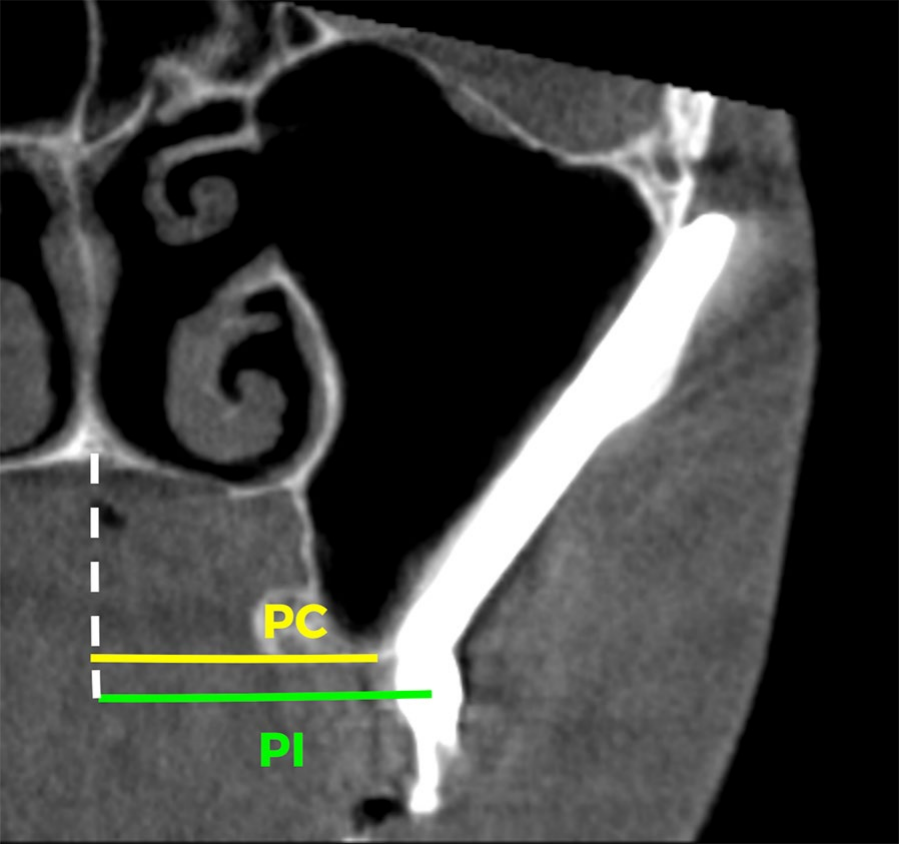
Distance from the palate half-way point to the crest midpoint (P–C) and distances from the palate half-way point to each implant head center (P–I) were measured. Distance P–C minus distance P–I indicates the relationship of the prostheses with the crest

**Fig. 7:**
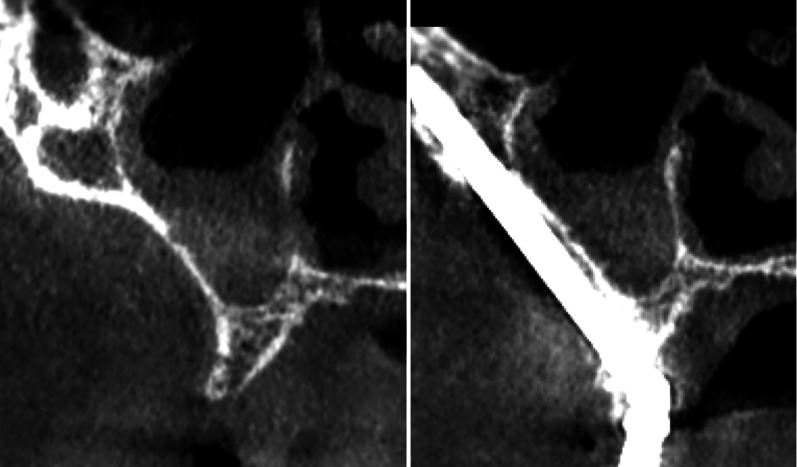
Preoperative CBCT images are compared to one-year postoperative images. Although there is postoperative sinus opacity, the Modified Lund–Mackay score [20] would be negative because the referred opacity existed before the surgery and did not increase afterward

In addition to recording possible complications during surgery and other complications common with regular implants such as a lack of integration or infections, the outcome—that is, the combination of the ZAGA protocols together with the new Straumann® Zygomatic Implant System—was evaluated using the ORIS criteria for a systematic report of ZI-related rehabilitation [20]. Four specific and objective criteria were used to evaluate the success of the rehabilitation:

1. Offset-evaluation of prosthetic success based on final positioning of the zygomatic implant with respect to the center of the alveolar crest: Using appropriate coronal cuts, distances (d) from the palate half-way point to the crest midpoint (P–C), and distances from the palate half-way point to each implant head center (P–I) were measured. Distance P–C minus distance P–I indicates the distance from the middle of the crest to the epicenter of the implant head. A positive value indicates a palatal position of the implant, whereas a negative value would indicate a buccal emergence of the implant [20] (Fig. 6).
2. Rhino-sinus evaluation: In 2014, Aparicio et al. suggested a system to report rhinosinusitis diagnosis in patients with zygomatic implants [21]. The system was revisited by Aparicio et al. in 2020 [20]. Data were reported in the same way as ear, nose, and throat (ENT) literature for conventional patients with some particularities. Essentially, sinus health after ZI installation was clinically and radiographically assessed following the Lanza–Kennedy (L-K) task force for rhinosinusitis clinical diagnosis [27]. The presence of clinical symptoms was used to grade the rhino-sinus situation of each patient over time. Patients received a Cone Beam Computed Tomography (CBCT) scan before surgery and again at one-year post-loading. Maxillary sinuses were examined by comparing both CBCT scans according to a modified Lund–Mackay (M L-M) score [20, 28] incorporated in the ORIS criteria (Fig. 7).
3. Soft tissue infection/inflammation evaluation: Any signs of infection or dehiscence were evaluated using a grading scale based on referenced photographs. In this regard, all subjects had photographic documentation of implant sites throughout the course of the study. The stability or progression of soft tissue recession was assessed together with the presence, or absence of, visual soft tissue inflammation or exudative signs (Fig. 4d).
4. Stability evaluation: When implant stability was checked, prostheses were unscrewed. Each implant was tested individually, accepting some mobility as a criteria for success. Dis-osseointegration, signs of rotation or apical pain were considered signs of failure.

Based on these criteria, one of five possible conditions were assigned (Table 2). Two blinded, independent researchers (B Al-N and WP) from different centers analyzed and classified the radiological and clinical results according to the ORIS criteria [20]. Analysis was based on the comparison of pre-operative and post-operative CBCT images and clinical pictures of the implant positioning, mucosal status, and occlusal prosthesis’s view.

**Table 2:** ORIS success levels

Level I: Success with optimal conditions
Level II: Complications without clinical impact
Level III: Borderline situation with complications that are clinically manifested but are still possible to successfully treat and resolve
Level IV: Not evaluated
Level V: Failure

## Results

### Patients

Twenty consecutive patients with indications for zygoma-related rehabilitation were treated. The first annual check-up included a CBCT. However, not all patients attended the annual check-up on time, as some of them did it later. Thus, the observation time calculated for this study is the time between the surgery and the last CBCT, regardless of the fact that other subsequent controls were eventually performed, which for ethical reasons did not include new CBCTs (Table 3). The first patient underwent zygomatic surgery in June 2019, and the last surgery was performed in October 2020. Once treated, patients were followed for a period between 12 and 28 months (mean follow-up 18,8 months) (Table 3).

**Table 3:** Patient population

Gender	Age	Smokers	Sinus floor discontinuity	Follow-up (months) Surgery to last CBCT
45% male 55% female	59.2 ± 8.4 ys	25.0%	20.0%	18.5 ± 5.2

Ten patients received 2 zygomatic implants plus regular anterior implants; 9 received 4 zygomatic implants and 1 patient received 3 zygomatic implants plus 2 regular implants on the anterior maxilla. In total, 59 zygomatic implants were placed, 36 Straumann® ZAGA-Flat and 23 Straumann® ZAGA-Round designs. Implant distribution by type and position is detailed in Tables 3, 4 and 5. Seven (35%) of the subjects were considered smokers. Four patients (20%) presented previous discontinuities of the sinus–nose floor (Table 3). Fifteen patients (26 sites) presented previous maxillary sinus opacities. Out of them, 1 patient (P-6) presented 2 sites on the same maxillary sinus that severely affected ostium patency.

**Table 4:** Types of zygomatic implant rehabilitation

Number of zygomatic implants used	Number of patients	%
2	10	50
3	1	5
4	9	45

**Table 5:** Implant design used as a function of ZAGA classification

ZAGA classification	Frequency (%)	Flat design (%)	Round design (%)
Type 0	3.4	0	100
Type 1	8.5	40	60
Type 2	27.1	18.8	81.3
Type 3	11.9	28.6	71.4
Type 4	49.2	93.1	6.9
Total	100	58	42

All implants reached more than 45 N.cm of insertion torque except four implants in the same patient. All implants were connected with screw-retained abutments suitable for the construction of screw-retained prostheses. All patients received an immediate screw-retained prosthesis within 4 to 24 h. After completion of follow-up, 100% survival rates of both the implant and prosthesis were noted in all patients.

### ORIS level

#### Prostheses offset (the “O” criterion)

**Fig. 8:**
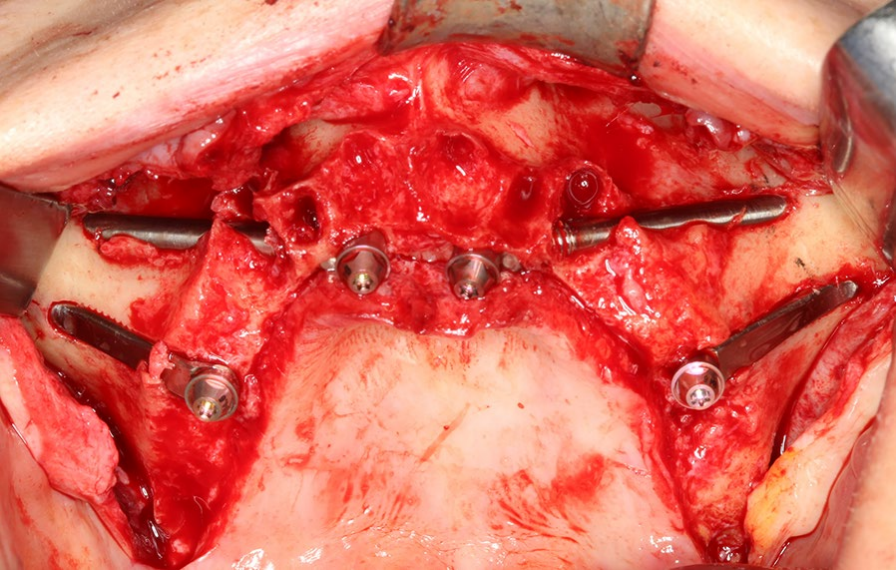
Occlusal view after the installation of two Straumann ZAGA-Round zygomatic implants on the anterior maxilla and two Straumann ZAGA-Flat implants on the premolar zone. Note that the ZAGA-Round implant is used to close a circular tunnel osteotomy; this is why the implant head is placed on the palatal side of the remaining crest. When it comes to the ZAGA-Flat design the implant head is located right on the middle of the crest

The center of the implant head was consistently located at the residual alveolar crest when the flat design was used; or at most, 4 mm palatal when the round design was used (Fig. 8).

**Fig. 9:**
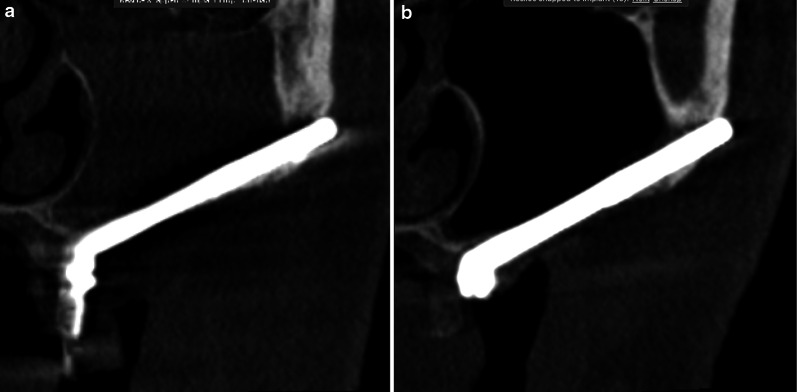
**a** The CBCT of patient 18 taken ten days after the surgery showed a positive M-LM score with almost complete maxillary sinus opacity and the clinical LK test was also positive. The patient was diagnosed with acute rhinosinusitis and treated with antibiotics and local corticosteroids. **b** The CBCT image of patient 18 taken 1 year postoperatively. Total transparency of the sinus was present; the M L-M score was negative. (In collaboration with Drs. Guy Mclellan and Ophir Fromovich.)

**Fig. 10:**
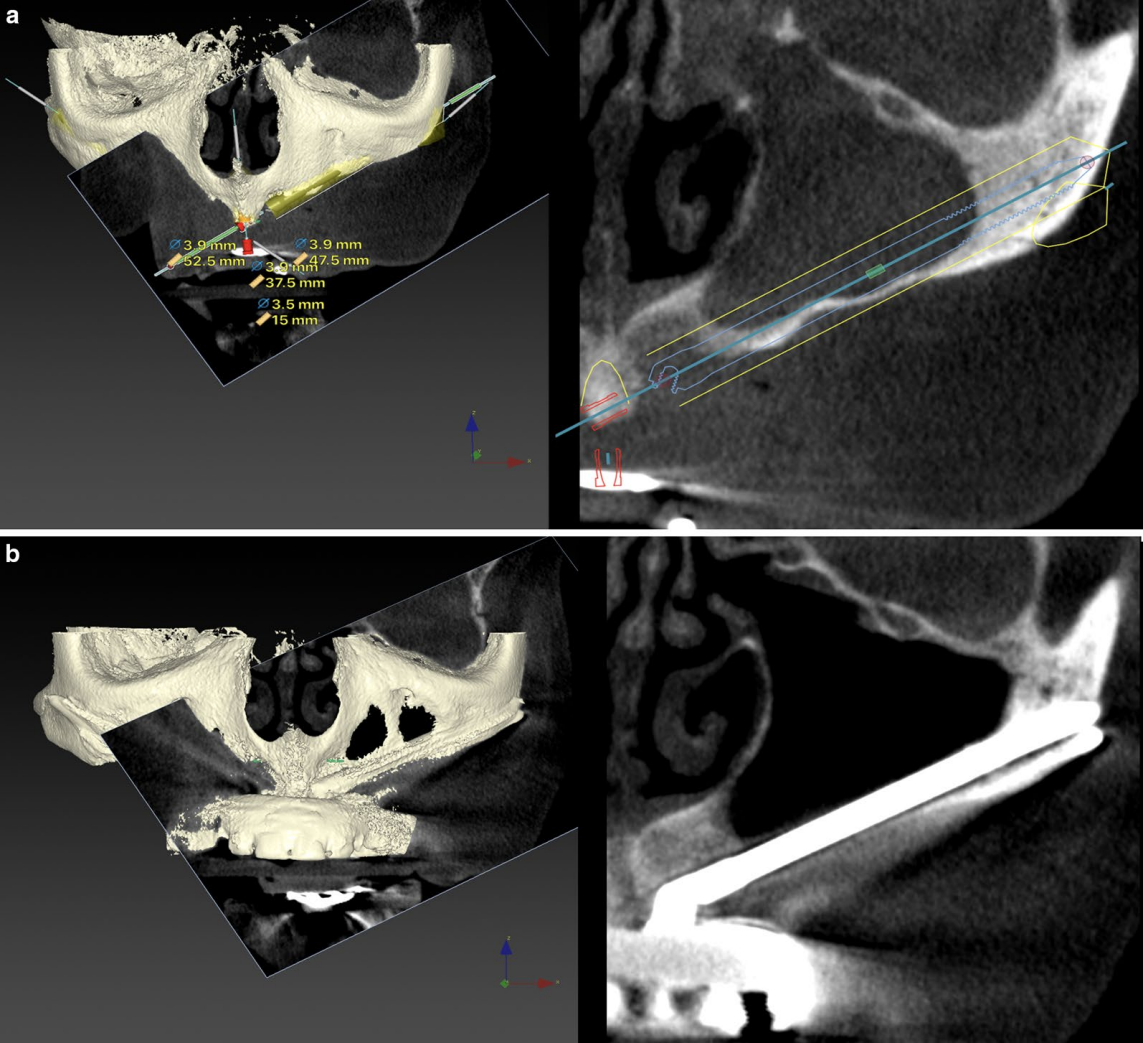
**a** Oblique pre-surgical CBCT cut of patient 6 illustrating the anterior left zygomatic implant planned trajectory. Note the bony defect under the nose caused by the previous loss of a regular implant. Note also that the maxillary sinus is totally occupied and the osteo-meatal patency is compromised. **b** One year postoperative oblique CBCT cut of patient 6 illustrating a total regression of the sinus occupation and recovered ostium patency

**Fig. 11:**
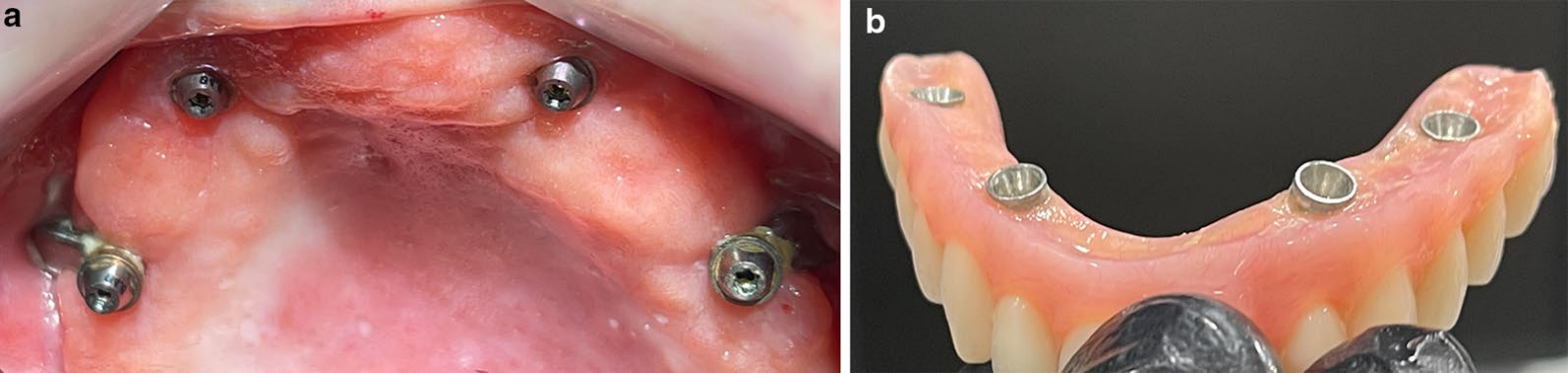
**a** Intraoral picture of patient 11. Soft tissue dehiscence was present on the two posterior zygomatic implants and not on the anterior ones. Although there is plaque accumulation the mucosa is not inflamed. **b** Gingival view of the immediate prostheses of patient 11. Note the excessive flange high touching the soft tissues and preventing appropriate hygiene

**Fig. 12:**
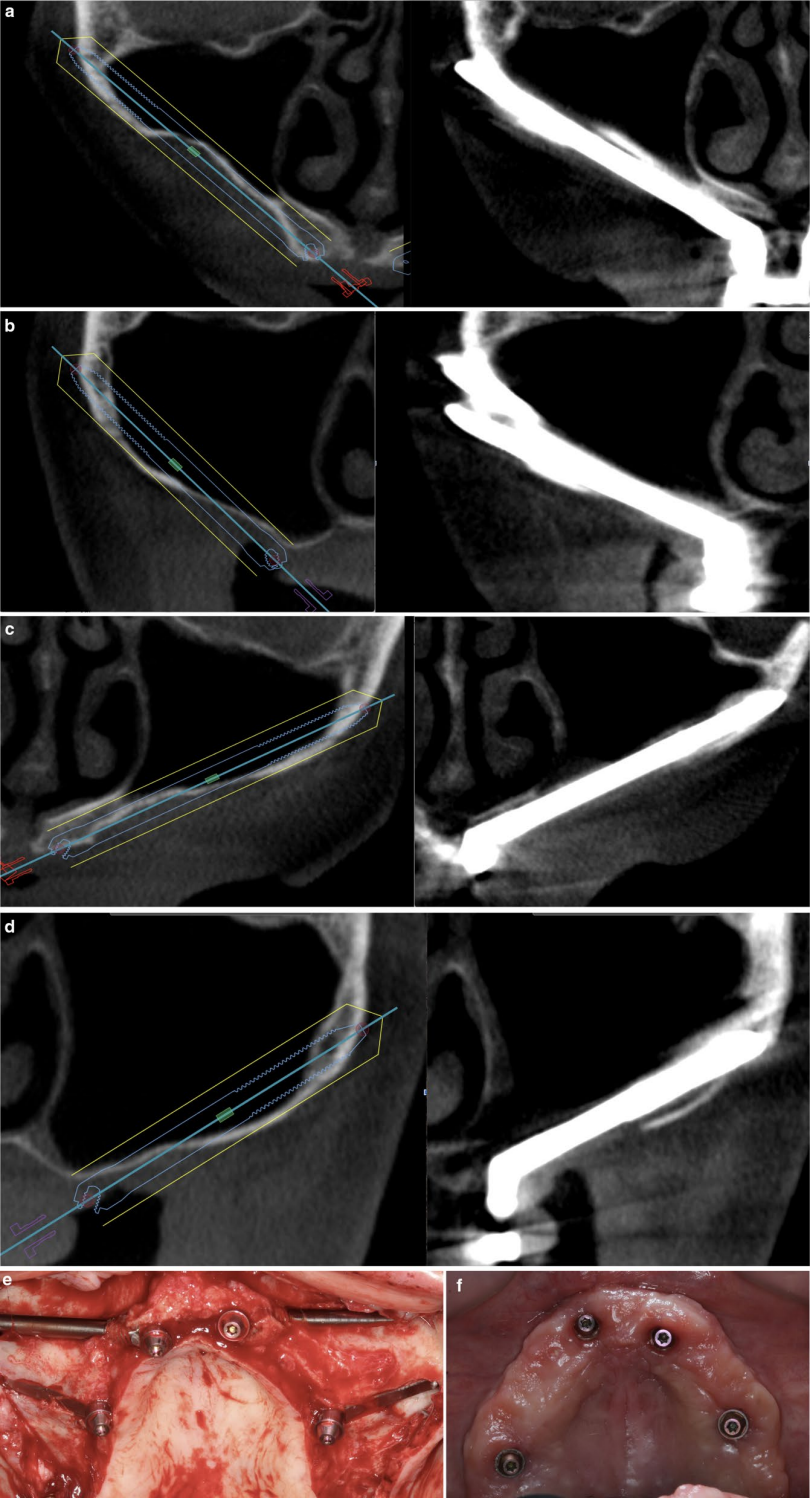
**a**–**d** The preoperative virtual planning in the extremely atrophic maxilla of patient 5 is compared with the postoperative one-year CBCT. **a** represents the anterior right implant comparison; **b** is posterior right; **c** is anterior left; **d** is posterior left. The implant placed in the position of the second left premolar showed intrusive movement with no associated rotation or pain. 26 months after the surgery the implant is still in function. The implant was classified as success grade III. **e** Occlusal intraoperative view of the patient 5. Two Straumann ZAGA-Round were placed on the anterior zone and two Straumann ZAGA-Flat on the premolar/molar zone. Note the two different types of osteotomy designed to protect both sinus integrity and soft tissues. **f** Occlusal gingival picture of patient 5 illustrating the routine soft tissue assessment necessary to understand and report whether or not the rehabilitation is a success

The evaluation of implant head emergence led to a “success level I” in all patients.

#### Rhino-sinus condition (the “R” criterion)

17 patients showed either transparency or non-increased maxillary sinus opacity when comparing pre-surgical with 1-year post-surgical CBCT scans. Indeed, 17 patients had (−) Modified Lund–Mackay.

Three sites in three different patients displayed a light increased opacity that did not affect ostium patency and had no clinical significance: three patients had ( +) Modified Lund–Mackay). Success level II”.

One patient (P18) revealed clinical and radiological signs of acute rhinosinusitis in the right maxillary sinus one week after surgery. This followed the placement of a ZAGA-Flat implant using a channel-type osteotomy. The symptoms disappeared completely after standard medical treatment. One year later, a new CBCT showed total transparency with no signs of mucosal inflammation. The left side, where an anterior ZAGA-Round and a posterior flat ZI were placed, remained asymptomatic and without signs of inflammation during the entire process (Fig. 9a, b). As a result, the patient was classified as (−) Modified Lund–Mackay.

17 sites in 11 different patients exhibited decreased or no opacity when comparing pre-surgical with 1-year post-surgical CBCT scans (Fig. 10a, b).

The Lanza–Kennedy (L-K) for rhinosinusitis clinical diagnosis was found to be negative at the one-year control in all patients.

The evaluation of the rhino-sinus condition led to a “success level I” in 17 patients to a success level “II” in 3 patients.

#### Soft tissue stability/infection (the “I” criterion)

Four implants showed non-inflammatory soft tissue alterations. In one patient, two flat design implants presented non-inflamed soft tissue recession together with inadequate prosthesis design (Fig. 11a, b). One round design implant showed recession with no inflammation despite signs of plaque accumulation, and in a heavy smoker, one round implant design showed a mucosal fenestration with no inflammation at the 18-month control.

The evaluation of soft tissue stability led to a “success level I” in 17 patients, to a success level “II” in 2 patients, and a “success level III” in one patient.

#### Implant stability (the “S” criterion)

Fifty-eight implants were considered stable. One implant placed in an extremely atrophic maxilla and zygoma showed slight intrusion movement but the patient felt no pain or rotational motion (Fig. 12). 29 months after surgery, this implant was still functioning.

The evaluation of implant stability led to a “success level “I” in 19 patients, and a “success level III” in one patient.

## Discussion

This prospective non-interventional study evaluated clinical outcomes of treatment with new zygomatic implants designs according to the ZAGA Concept. Indeed, all zygomatic implants in this study were placed following the ZAGA Concept and, using site-specific zygomatic implants.

As there were no intraoperative complications, in the analysis of the behavior of the new implants more emphasis on late complications specific to the zygomatic implants has been given. The few late complications that did appear could not be related to the type of ZAGA implant used. The choice of either one implant design or another is made in relation to a specific anatomical situation. Consequently, the occurrence of a complication related to the implant design also occurs in different, and not comparable situations.

ORIS criteria allowed clinicians to follow up with their patients using standardized success criteria and to objectively evaluate the prosthetic aspect, the maxillary sinus status, the peri-implant soft tissue condition, and also, zygomatic implant stability [20].

Results revealed that the healing process was uneventful, with none of the implants showing relevant clinical problems including fistulas The ZI survival rate of 100% found in this study is consistent with other studies using a similar clinical follow-up. A recent meta-analysis reports a survival rate of 98.35% for the follow-up of 6 to 12 months [22] while a systematic review on immediate loading ZI reports a survival rate range of 96–100% [29].

In this study, the use of the Lanza–Kennedy clinical task force and Lund–Mackay radiological score should be noticed because they provide a standardized system to classify sinus status. Fifteen patients (26 sites) presented previous maxillary sinus opacities, 1 patient presented preoperative opacity in 2 sites severely affecting ostium patency, and four patients (20%) presented previous discontinuities of the sinus–nose floor. However, just 1 patient experienced immediate postoperative sinusitis, and it was clinically and radiologically solved with medical treatment. Seventeen patients showed either transparency or non-increased maxillary sinus opacity when comparing pre-surgical conditions with 1-year post-surgical CBCT. Three sites in 3 different patients displayed a light increased opacity with no clinical significance. The authors have no explanation for the fact that 17 sites in 11 different patients exhibited decreased or no opacity when comparing pre-surgical with, at least, 1-year post-surgical CBCT scans (Fig. 10a, b). This is probably related to the anatomically guided way of placing zygomatic implants together with the use of site-specific ZAGA implant designs. However, this theory should be further investigated in a longer follow-up setting.

The results of the present study seem to be in contradiction with the results obtained by Zhao's group whereby after the insertion of 84 zygomatic implants, a thickening of the membrane was observed over time. This is in addition to a noticeable decrease in the permeability of the ostium [30]. Yet, according to Zhao and coworkers, the ZAGA protocol was followed where they describe the use of a slot in the maxilla prior to implant placement. Additionally, they do not give clear data for the choice of the location of the zygomatic implant critical zone. These are deviations in the ZAGA surgical protocol and they could explain the differences in results between the two studies in terms of post-surgical thickening of the sinus lining or decreased patency of the ostium.

Routine soft tissue assessment is necessary to understand whether or not the rehabilitation is a success. In this regard, it is important to describe not only the presence of a recession but also the stability of such a recession over time. The presence of associated mucosal infection and related esthetic problems are also key parameters to be analyzed. All patients involved in the study were examined and assessed on the quality of the soft tissues surrounding the zygomatic implants according to ORIS criteria in a standardized manner. Although being a common clinical complication in the long term, soft tissue recession is sporadically reported in peer-reviewed publications [31, 32].

In a recent multicenter study following the same clinical approach, the vast majority of implants were placed extra-sinus or in the wall of the maxilla (ZAGA II–IV), and just a minority were placed in an intra-sinus position [16]. This seems to be similar to the situation found in this study. This reflects the anatomical situation of the patients presenting maxillary bone atrophy. It also demonstrates that the placement of zygomatic implants by means of the ZAGA approach may avoid the sinus space in most cases.

All implants included in this study were loaded immediately, i.e., within 48 h of placement. Application of the immediate loading protocol was enabled by the general achievement of high primary stability, as measured by the final insertion torque. The authors attribute this to both the tapered design of the zygomatic implants and the minimally invasive under-preparation of the implant sites.

Fifty percent of the sites were classified as ZAGA type 4, and 27 of the sites were classified as ZAGA type 2. In addition, 20% of the patients had discontinuities in the floor of the sinus or nose or both. This demonstrates the fact that most of the patients treated in this study had extreme maxillary atrophy. Patients with severe or extremely severe bone resorption were treated using quadruple zygoma installation. In those patients, the use of narrow tapered implants and the ZAGA concept enabled immediate loading protocols without prior regenerative approaches. Consequently, the patients were provided with a fixed provisional prosthesis, thereby shortening time-to-teeth, especially when compared to alternative bone grafting procedures. Immediate implant loading, as performed in this study, is a valuable treatment option because it offers the patient an immediate restoration of function, esthetics, and social confidence [3].

Subjects included in controlled clinical studies are selected based on strict inclusion and exclusion criteria and may not necessarily reflect the normal patient population found in a dental practice [24]. In this study, no exclusion criteria regarding tobacco consumption or sinus status, other than acute infection have been used. Indeed 75% of all treated patients presented partial or even total sinus occupancy, 25% were heavy smokers and 20% of them had bony defects/discontinuities at the sinus or nasal level. For these reasons, it has been argued that the results of clinical studies may not be representative of actual outcomes seen in a general population, thus indicating the need for additional observational studies [23]. Although there is more freedom in treatment decisions with an observational study, the systematic documentation of the device’s usage and clinical outcome provides an overall assessment of the device’s effectiveness [24]].

## Conclusion

Within the confines of patient limitation, a non-interventional study design, and a follow-up period of 12 to 28 months (average 18,8 months), the rehabilitation of the atrophic maxilla by means of new ZI designs resulted in a low complication rate, even in extreme anatomical situations like the ones accepted in this study. Moreover, the 100% survival rates of both the implant and prosthesis indicate that the use of ZAGA-Flat and ZAGA-Round zygomatic implants following the ZAGA protocol offers a viable treatment option when restoring the extremely resorbed maxillae.

## Data Availability

The data sets generated and analyzed during the present study are available from the corresponding author on reasonable request.

## Abbreviations

ZAGA: Zygoma anatomy-guided approach
CBCT: Cone beam computed tomography
ORIS: Offset (prosthetic); Rhino-sinus (status); Infection/inflammation (soft tissues); Stability (implant)
P–C: Distance from the palate half-way point to the crest midpoint distances
P–I: Distance from the palate half-way point to implant head center
ENT: Ear, nose, throat
L-K: Lanza–Kennedy task force for rhinosinusitis clinical diagnosis
L-M: Lund–Mackay score
M L-M: Modified Lund–Mackay score
